# Biochemical Refinement Before Genetics: Chance Utility

**DOI:** 10.1007/s00239-016-9757-5

**Published:** 2016-09-01

**Authors:** Michael Yarus

**Affiliations:** Molecular, Cellular and Developmental Biology, University of Colorado, Boulder, Boulder, CO 80309-0347 USA

**Keywords:** Random, Evolution, RNA, Selection, Near-ideal

## Abstract

Given two primordial conditions that seem likely to be common, near-ideal reactions for evolutionary progress are realized. These requisites are sporadic availability of pooled reactants and evolutionarily useful products within a pool’s repertoire. These intrinsically optimizing circumstances function without genetics, and therefore can help evolve a first genetic system. This process is termed chance utility.

## Utility in Chance Events

A pool is a locus where geochemicals collect, and may react. A sporadically fed pool further embodies hypothetically chaotic primordial conditions. It receives chemicals undependably, at random times (exponential interval distribution), and in undependable, varying (normally-distributed, ≥0) amounts (Yarus 2012). A type of recurrent biochemical success has emerged in studies of sporadically fed ribonucleotide pools (Yarus 2013). Here I illustrate this recurrent successful behavior using a sporadically fed pool that avoids a potentially obstructive pollutant.

## Evasion of a Poisonous Reactant

Consider a reaction poison which consumes a required substance by forcing a side reaction. For simplicity, *A* and *B* yield the potentially useful biochemical *C*

1 $$A + B \to C.$$

An alternative poison reactant, *P*, consumes the common reactant, *A*

2 $$A + P \to Q$$

via a similar reaction to yield (hypothetically useless) *Q*. In this way, *P* prevents synthesis of the potentially advantageous *C*. All reactants are assigned reasonable relative stabilities for nucleotides (Yarus, submitted): *A* and products are stable (as are pN) and *B* and *P* are equally unstable (as are activated pN). To make the outcome more transparent, the desirable reaction (1) and the poisoned reaction (2) have the same rate constants (see the Figure legend).

**Fig. 1:**
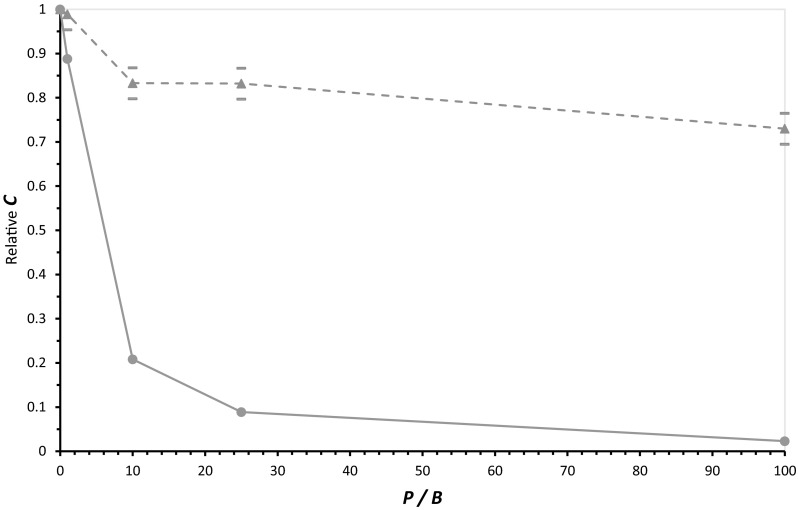
Inhibition of *C* production at different ratios of poison *P* to reactant *B*. The upper line (*triangles, dashed line*) plots mean relative yield of *C* in 100 examples of a sporadically fed pool; the lower line (*circles, solid*) plots relative *C* (inhibited/control reactions) in a more typical incubation in which all reagents are combined initially, and held until time = 100. For the sporadically fed pool: Reactants arrive randomly, but at an average of 10 times/100 days. For both reactions: Decays are first order: *B* and *P* decay at 1/day, *A* decays at 0.01/day. Products decay at 0.001/day. Reagent *A* and *B* arrivals are of magnitude 0.001 ± 0.0005 M (SD), poison *P* arrivals are set at none to 0.1 ± 0.05 M (SD). The second order rate constant for *A* **+** *B* and *A* **+** *P* reactions is 1000/M/day. *Bars* above and below the points represent the standard error for each mean of 100 simulations

In Fig. 1, when *A* and *B* are combined and incubated for 100 days, the result is a calculable level of the useful product *C*. However, *A* and *B* reactions can also be initiated with the poison *P* added, which reduces the accumulation of *C* (Fig. 1, circles, lower plot). The Figure compares this to a sporadically fed pool (triangles, upper plot) receiving *A*, *B*, and *P* at sporadic times. Poison *P* is present at 0–100-fold the concentration of its alternative, competitive reactant *B*.

Figure 1 quantitates poisoning. When *P* is in greater molar excesses to *B*, the yield of the simultaneous *A* **+** *B* reaction declines. As intuition suggests, when P is in 100-fold excess (reaction of *A* with *B* and *P* have the same rates), production of *C* is almost completely prevented (*C* is about 2 % that in the absence of *P*; see the lower right of the Figure).

Notably, however, when the same reagents appear in a sporadically fed pool (upper dashed curve) instead of a normal laboratory-style reaction, 100-fold excess of poison *P* only decreases *C* to about 73 % that without poisoning. Even large amounts of poison, therefore, have little consequence for the evolutionary desirable outcome in a sporadically fed pool.

**Fig. 2:**
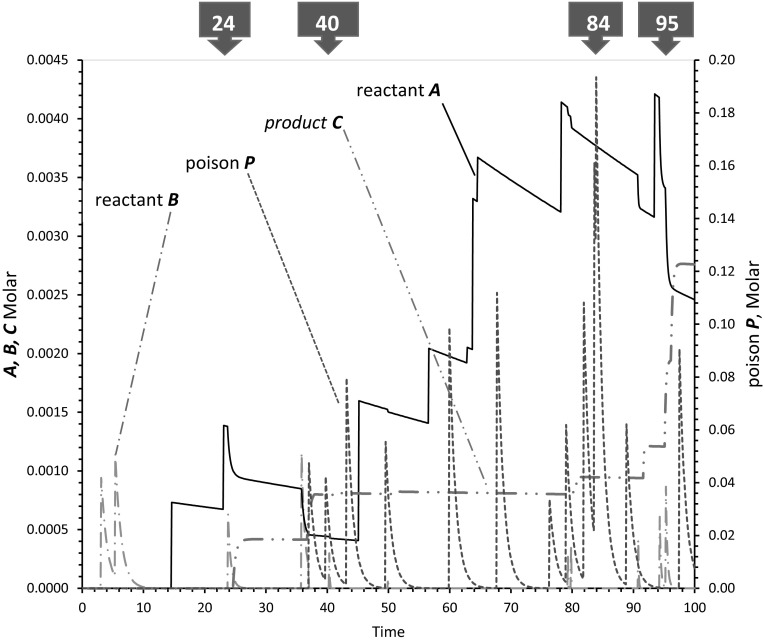
Molar concentrations versus time in a representative, maximally poisoned, sporadically fed reaction. Stabilities and reaction rates from the text were implemented and numerically integrated 1000 times/day for 100 days using the Rosenbrock integrator of Berkeley Madonna v. 8.3.23.0 (Yarus 2012), and resulting product concentrations were processed in Microsoft Excel 2013. Times in *numbered boxes* at the top tag characteristic events discussed in the text. Rate constants are the same as in Fig. 1

Figure 2 shows why the sporadically fed pool is unexpectedly resistant to poisoning, by plotting concentrations in a pool receiving *A*, *B*, and *P* sporadically. Data follow a representative example of the most poisoned reaction, with spikes of poison *P* in 100-fold excess over *B*. Synthesis of *C* (dashed and dotted line) is resistant to alternate reactant *P* because *C* synthesis occurs in isolated episodes when *A* (solid line) and *B* (dashed and double-dotted) are present together, as at 24 days (under the tag marked 24). After the *B* spike at 24 days, both *B* (dot and dash) and *A* (solid line) are consumed to yield *C* (double dot and dash). Inhibition by *P* is possible: note that production of *C* is poisoned at around 40 days (upper tag marked 40). Or at 79 days, potential synthesis of *C* is greatly reduced by reaction with *P*. But these inhibitory events are atypical.

This pool would be expected to, on average, accumulate its product *C* nearly proportionate to the square of pool age (Yarus, submitted). In accord with this prediction, *C* appears mostly in late events. The major late production of *C* due to adjacent spikes of *B* at 94–96 days (upper tag marked 95), which account for the majority of total *C* synthesis, occurs in the clear between flanking spikes of *P*. Appearance of *C* is therefore substantially unimpeded (Fig. 2). In fact, for about 5 days (92–97 days) *C* as well as its precursors, *A* and *B* are available to a possible evolutionary descendant in the near-complete absence of poison. Just before this availability, poison *P* almost doubles its own standard concentration by chance superposition (tag marked 84), but without malign effect.

One can express these quantitative kinetic results as follows. A prudent biochemist would likely not claim that the 1 % contaminant, *B*, was the significant reactant in our scenario, because 100 times as much of an equally reactive competitor *P* is present. But as Figs. 1 and 2 suggest, *B*’s importance is plausible. And *B*’s successes are not rare, but recur routinely, as shown by Fig. 1’s averages of 100 pools (triangles, dashed line). Although 100-fold molar excesses of poison *P* can be a formidable obstacle to *C* synthesis (circles in Fig. 1), this obstacle is readily by-passed in the pool. This is chance utility.

## Other Examples

Not only have other pools worked similarly, but favorable chemical sequences of surprising complexity are emphasized via chance utility. In recognition of these creative outcomes, I previously noted that a sporadically fed pool can maximize its output by assembling a near-ideal series of reactions (Yarus 2013).

## Elevated Substrates

For example, pool output selectively utilizes high concentrations of substrates that recur because of accidental overlaps in random nucleotide supplies (Yarus 2013). This accident strongly elevates reactants above the standard supplied concentrations.

## Favored Pathways

Under the same conditions, a sporadically fed pool containing potential replicators produces its product in the subset of random nucleotide spike sequences which encourage a specific, optimal sequence of reactions: first favoring synthesis of a template, then supporting replication of the newly appeared template. In fact, in this case one can make an even stronger claim; almost all the output from pool chemistry comes from the near-ideal subset of reactions (Yarus 2013, 2012).

## Extended Reaction Sequences

Moreover, unexpectedly, complex sequences of events can occur because the more stable products of early reactions persist to present a large target for reaction with later random arrivals of nucleotide reactants (Yarus 2013). The result is an unexpectedly high frequency of reaction chains employing, for example, eight or more random reagent arrivals in support of a single product.

## Accumulation of Stable Reactants

In a sporadically fed pool specifically containing cross-templating ribonucleotides (Puthenvedu et al. 2015; Majerfeld et al. 2016), mean output from the pool is ultimately dominated by exceptionally efficient templating events that utilize unstable reactants efficiently by accumulating more stable substrates (Yarus, submitted). The result of these efficiencies is that pool products accumulate as the third or fourth power of pool age (Yarus, submitted), frequently giving encoded products of the same order of concentration as nucleotide precursors.

## Generalized Chance Utility

Particular examples cited above differ in detail, but share a common logic. Call this shared pattern chance utility, to emphasize that success comes directly from chance events that define a sporadically fed pool. That is, random supplies of substrate imply that all successions (and all amounts) of reagents will be tested. Effective reactant sequences produce product, the ineffective do not. Thus when pool outputs are selected quantitatively, it will necessarily be found that success is predominantly due to particular near-ideal reactions (Yarus 2013). Remarkably, and to an extent that is obscure until the calculations are done, this implies that favored unguided pools, with randomized supplies of reagents, are selectively producing output from the (possibly small) class of optimal pool reactions. Near-optimal syntheses from random repetitions in one or a group of pools are what is meant by chance utility. Such optimization is a fundamental reason that a sporadically fed pool is uniquely suited to origin functions. Our opening poison example illustrates this by offering (at 95 days) large amounts of useful product *C*, alongside its precursors, when no poison *P* need accompany it into a descendant.

It is important that chance utility is not tied to very restricted circumstances. Whenever substrates arrive in an erratic manner, possible reaction sequences are surveyed. For example, the rigorous exponential interval distribution between substrates in the sporadically fed pool (Yarus 2012) is therefore not mandatory. Whenever particular results favor a successful evolutionary sequence, these results are more likely to be incorporated into descendants and preserved. Therefore it is a serviceable anthropomorphism to think of a chaotic pool as searching its limited chemical repertoire for evolutionarily optimal results. In spite of the substantial number of quantities that must be specified to explicitly calculate what a pool of cross-templating RNAs will probably do (Yarus, submitted), none of this quantitation is, in the end, essential to chance utility. Given only varied chemical opportunities and a selection for successful chemical outcomes, chance utility fulfills its blind search.

Chance utility can usefully be compared to natural selection (Wallace 1858; Darwin 1859). In both processes, selection among variants can yield progressive improvement. But for chance utility, the variation is not genetic, though simple genetic phenomena can themselves be selected via chance utility in pools (Puthenvedu et al. 2015; Majerfeld et al. 2016; Yarus, submitted). Pool variation comes from inevitable chemical differences, even in a constant environment (Yarus 2012, 2013, submitted). The poison example set out above contains no genetic phenomena, though it exploits pool fluctuation. Hence, pool successors can possess nonrandom chemical assemblies prior to genetics, formed via chance utility.
